# Maturation of gastric electrical activity, gastric emptying and intestinal permeability in preterm newborns during the first month of life

**DOI:** 10.1186/1824-7288-35-6

**Published:** 2009-03-15

**Authors:** Giuseppe Riezzo, Flavia Indrio, Francesco Raimondi, Osvaldo Montagna, Gennaro Salvia, Bisceglia Massimo, Lorenzo Polimeno, Luciano Cavallo, Ruggiero Francavilla

**Affiliations:** 1 Laboratory of Experimental Pathophysiology, National Institute for Digestive Diseases IRCCS, Saverio de Bellis, 70013 Castellana Grotte, Bari, Italy; 2 Department of Pediatrics, University of Bari Policlinico, Piazza G Cesare, 70124, Bari, Italy; 3 Department of Pediatrics, University Federico II Policlinico, Via S Pansini 12, 80100, Naples Italy; 4 Department of Pediatrics, Ospedale Fatebenefratelli, 80100, Naples, Italy; 5 Department of Pediatrics, Ospedale Sangiovanni di Dio, 21009, Crotone, Italy; 6 Department of Organ Transplantation, Division of Gastroenterology, 70124 University of Bari Policlinico, Piazza G. Cesare, Bari, Italy

## Abstract

**Introduction:**

Immaturity of motility, intestinal epithelial barrier function and absorptive capacity may play a role in the pathophysiology of intestinal diseases in preterms. We determined the gastric electrical activity and emptying, and intestinal permeability, in preterm newborns to verify if a maturation pattern exists in preterm newborns during the first month of life.

**Patients and methods:**

Eighteen preterm newborns (median 34 wks, range 2 wks) completed the study. They underwent the recording of gastric electrical activity by means of cutaneous electrogastrography, the ultrasound examination of gastric emptying, and the lactulose-to-mannitol ratio from permeability-absorption test on days 3, 7, 15, and 30 after birth.

**Results:**

Gastric electrical activity and emptying showed only slight changes between day 3 and day 7. On the contrary, an evident maturation in permeability, expressed as L/Mratio, was evident over time (Friedman Repeated Measures Analysis, p = 0.004).

**Conclusion:**

In preterm healthy newborns of 34 weeks gestational age, electrical and motor activity are completely developed at birth whilst the intestinal epithelial barrier clearly improves during the first week of life.

## Introduction

Feeding intolerance is a recurrent problem in the clinical care of preterm infants and occur mainly in the first week of life, suggesting the presence of a maturation pattern of gastrointestinal tract [1]. It is known that functional maturation of the gastrointestinal tract is quite different over time with respect to its anatomical development [2-4]. Adequate levels of some digestive enzymes are reached only at the end of gestation and lactase activity at 34 weeks gestation is only 30% of the level of full-term newborns [3]. To date there is little data available about the development of the motility function and of the mucosal barrier in newborns during early days of life.

Gastrointestinal motility can be recorded as a measure of gastric electrical activity, of the wall movements, and of gastric emptying time. A reliable method for recording gastric motility is cutaneous electrogastrography (EGG) [5-7]; electrogastrographic studies in newborns have demonstrated the absence of normal slow waves at birth and a maturation process modulated by enteral feedings [8-11]. Gastric emptying (GE) can be assessed by ultrasonography which is considered a non-invasive technique particularly suitable for young patients [12].

The functional integrity of the mucosal barrier of the intestine partly depends on the close interaction of adjacent mucosal cells. The most reliable in vivo method to study this functional integrity is the sugar absorption test (SAT), which has been performed on adults [13] and newborns, both preterm [14] and term ones [15]. Some of the key events involving permeability actually take place in the neonatal period, when the barrier is leakier. Coordinated motor function in the gastrointestinal tract plays a crucial role in the intestinal transportation, absorption and maintenance of the enteric bacterial ecology [16]. In particular, delayed intestinal transit time may contribute to increased mucosal permeability, and even to facilitated bacterial translocation [17].

The aim of the study was to investigate gastric motility and intestinal permeability to verify if a maturation pattern exists in preterm newborns during the first month of life.

## Methods

### Infants and protocol

The study was performed at the Neonatology Section of the Department of Pediatrics at the University of Bari. Healthy preterm newborns, born at a gestational age of 28–36 weeks, a birth weight > 1800 g, normal Apgar score, and a post natal age < = 24 h, were eligible to participate in the study. Newborns with: a) respiratory distress, b) congenital malformation, c) inborn errors of metabolism, or d) proven sepsis or infection, were not included. From an initial group of 38 preterm newborns, 18 entirely bottle-fed infants completed the study. The others were excluded for various reasons: a change in milk formula (4 newborns); an infectious disease (1 newborn); withdrawal from the study (7 newborns); inability to perform the scheduled SAT due to early transfer to another hospital (1 newborn) and/or failure to collect urine within the scheduled collection day (7 newborns). All the newborns enrolled reached the total amount of enteral feeding within the first week of life. All the preterm newborns were exclusively bottle-fed with the same preterm standard formula throughout the intervention period. The daily formula intake was approximately 30 ml/kg/day at baseline and 180 ml/kg/day at the end on the study.

Gastric electrical activity, gastric emptying time and intestinal permeability were recorded on days 3, 7, 15, and 30 after birth in order to evaluate the time changes in motility and permeability. The range of the data collection period was rigorously narrow (± 1 day). From birth until the end of the study, episodes of regurgitation, vomiting, number of evacuations, the time of complete emission of meconium, and the daily amount of feedings, were recorded. Written informed consent was obtained from the parents, and the study was approved by our local institutional ethics committee.

### Assessment of gastric electrical activity

The EGG recordings were performed using portable equipment before and 120 min after meal, following a fasting period of 4 hours. Two silver-silver chloride bipolar electrodes (Clear Trace, ConMed, Utica, NY USA) were placed on the cleaned abdominal surface overlying the antro-pyloric axis to obtain the best signal-noise ratio. The reference electrode was placed to form an equilateral triangle [18]. Electrogastrography was performed using a portable EGG recorder (UPS 2020, Medical Management Systems, MMS, The Netherlands). The recordings and analysis of the EGG parameters (dominant frequency and normal slow wave percentage, power ratio) were previously described in different papers [11,19]

### Assessment of gastric emptying

The ultrasound gastric emptying examinations were always performed by the same investigator using a real-time apparatus (Image Point HX, Hewlett Packard Company, Palo Alto, CA, USA) equipped with a 3.5 MHz linear probe. The ultrasound examination and the measure of the antral area were performed according to the procedure reported in a previous work [11]. The EGG and GE were simultaneously recorded to avoid differences due to the rapid changes in physiological parameters. During the same EGG recording session, antral measurements were made before the test meal, and at regular 30-min intervals up to 180 min after the meal. In each patient, the half emptying time (T1/2) was calculated [11,12,20].

### Assessment of intestinal permeability

The SAT was performed after oral ingestion, by suckling, of a solution containing 5.0 g of lactulose and 2.0 g of mannitol (Sigma Aldrich s.r.l., Milano, Italy) per 100 ml water (375 mosml/l) at a dose of 2 ml/kg of body weight. The newborns fasted two hours before and after the oral administration of the solution. All the urine passed in the subsequent five hours was collected in an adhesive urine bag (Sincrolag s.r.l. Italy). The complete urine volume was measured and stored at -80°C until analysis. Urinary concentration of lactulose and mannitol were determined by ion exchange chromatography with pulse amperometric detection [21]. Lactulose is a disaccharide that crosses the intestinal epithelium by passive diffusion through the paracellular tight junctions. Mannitol is a monosaccharide that crosses the intestinal epithelium mainly by transcellular passive diffusion through aqueous pores [22]. The evidence of an exclusively transcellular permeation of monosaccharides is still controversial. Mannitol is used for osmotic shrinkage of membrane vesicles, which would not be possible if permeation across cell membranes were unrestricted, and many experimental physiologists use it as an extracellular fluid volume marker suggesting a paracellular route of permeation for this probe [23].

The urinary excretion percentage of lactulose and mannitol are markers for paracellular and transcellular diffusion respectively. To correct for non-mucosal factors that may affect the intestinal uptake of these saccharides, including rate of gastric emptying, intestinal transit time and renal clearance, the urinary percentage of lactulose and mannitol were expressed as the L/M ratio.

### Data analysis

The data were first analyzed using simple descriptive statistics of centrality and dispersion. Clinical parameters are expressed as median and range and physiological data are expressed as mean ± SEM. However, because of the sample size and absence of a normal distribution of the data, only non-parametric statistical analysis tests were performed. The overall effect over time of EGG, GE and SAT parameters was determined by a repeated measures analysis (Friedman Friedman Repeated Measures Anova). Because of missing data at some points, multiple comparisons were non available and differences among the recording points of EGG, GE and SAT parameters were made using the Wilcoxon signed rank test. All the differences were considered significant at a 5% level. The software package used for the statistical analysis was STATA (STATA ver 4.0 Statistical Software, Stata Corporation).

## Results

### Anthropometric and clinical parameters

These parameters are reported in Table 1. It clearly shows that a homogeneous group was collected. None of the newborns presented significant regurgitation and/or vomiting from birth until the last day of examination and all passed the meconium within the second day of life. All newborns reached the total amount of enteral feeding (140 ml/kg/day) within the seven days.

**Table 1:** Baseline anthropometric and clinical data of the newborns which completed the study (n = 18)

Gestational age	34 [2]
Birth weight (g)	2140 [305]
Apgar score	8 [1]
Male/Female	8/10
Vaginal/caesarian delivery	7/11

Data are expressed as Medians and range or numbers

### Electrogastrographic and Gastric emptying data

**Figure 1:**
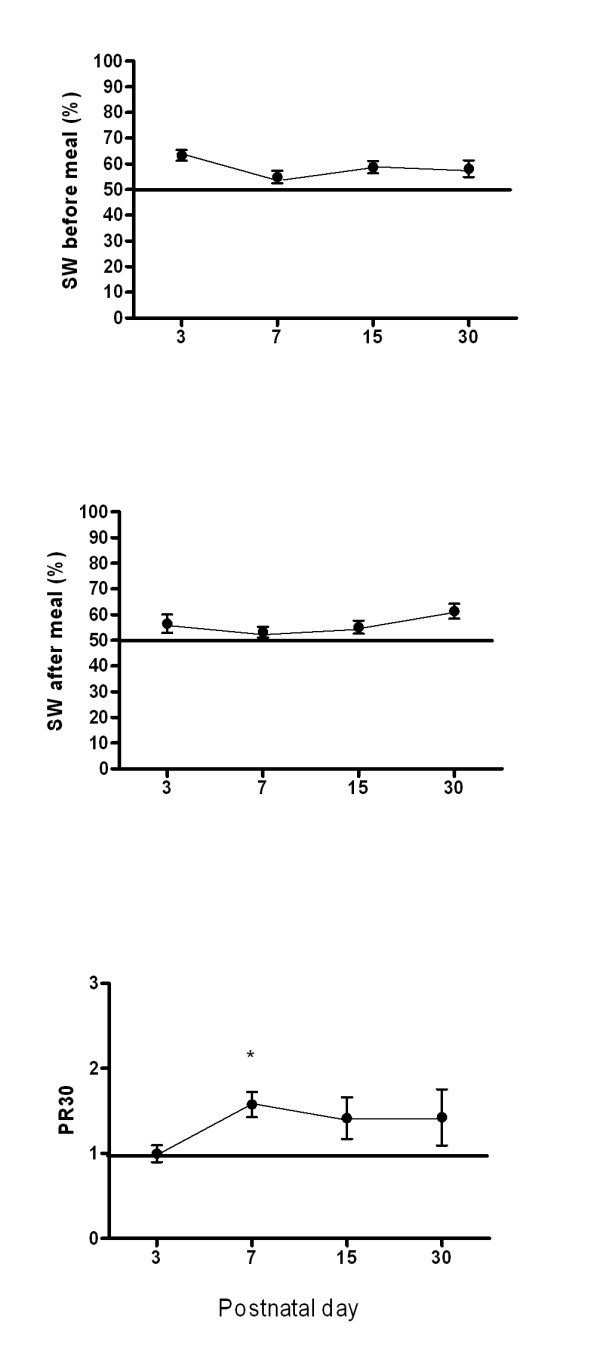
**Gastric electrical activity are reported as percentage of gastric slow waves (SW) at baseline (a), after meal (b), and power ratio (PR) (c)**. Repeated measurements analysis did not demonstrate any improvement in power ratio over time. Only a difference at day 7 respect to day 3 is evident. Data are means ± SEM.

**Figure 2:**
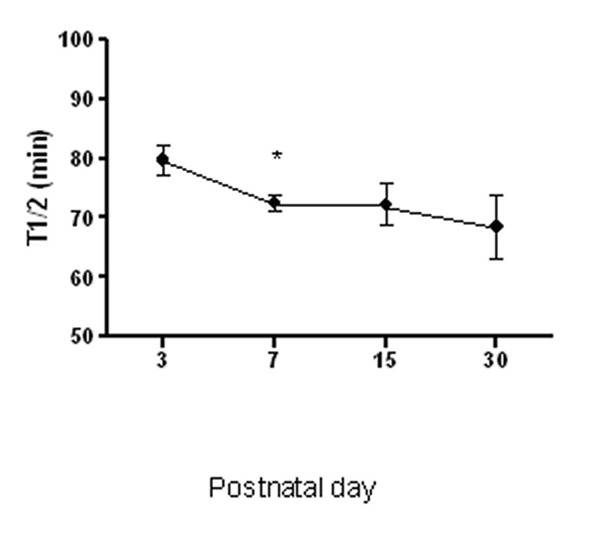
**Gastric emptying time is reported as the half emptying time (T1/2)**. Repeated measurements analysis did not show any improvement in T1/2 over time. A slightly difference at day 7 respect to day 3 is only evident. Data are means ± SEM.

Figure 1(a, b, c) shows the pattern over time of the percentage of normal slow waves recorded before and after meal, and the pattern of power ratio (Power ratio: Friedman Repeated Measures p = 0.18; Wilcoxon signed rank test, day 3 *vs *day 7 p = 0.02). Figure 2 plots the ultrasound T1/2 over time (Friedman Repeated Measures Analysis p = 0.69; Wilcoxon signed rank test day 3 *vs *day 7 p = 0.08). Both power ratio and gastric emptying time did show a slightly difference comparing day 3 and day 7, without a significant improvement over time.

### Intestinal permeability data

**Figure 3:**
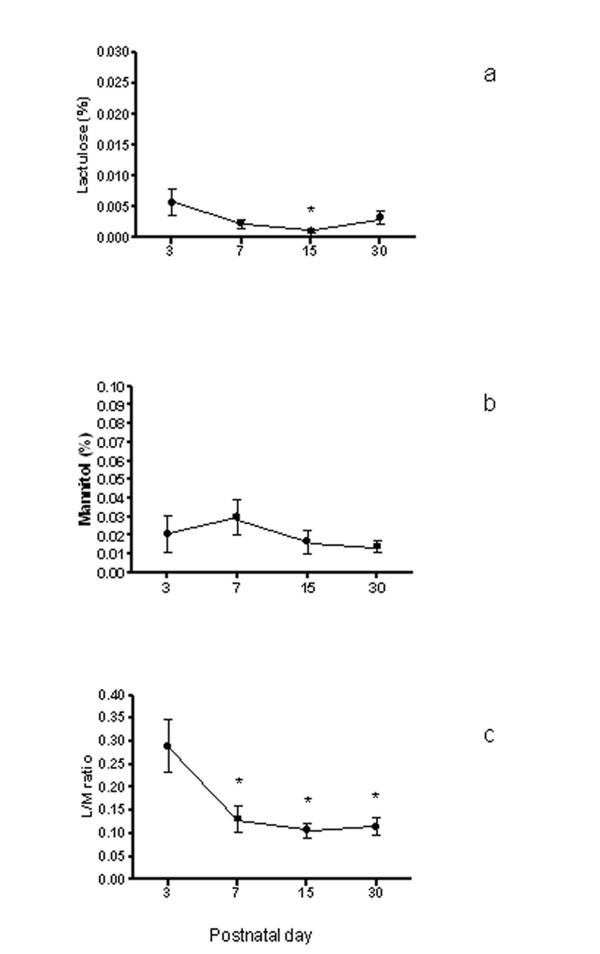
**Intestinal permeability pattern as determined by urinary excretion of orally administered lactulose (a), mannitol (b), and L/M ratio (c) respectively**. L/M ratio persistently and significantly reduces after day 3 (see text). Data are means ± SEM.

Measurement of lactulose excretion demonstrated an evident reduction at day 7 and the subsequent recording days (Figure 3a). On the other hand, measurement of mannitol excretion demonstrated a fluctuation over time and an increase on day 7 without reaching a significant difference (Figure 3b). The L/M ratio showed a deep decline between day 3 and day 7, then the ratio became constantly low (Friedman Repeated Measures Analysis p = 0.004; Wilcoxon signed rank test: day 3 *vs *day 7 p < 0.05, day 3 *vs *day 15 p < 0.05, day 3 *vs *day 30 p < 0.05 (Figure 3c).

## Discussion

In preterm newborns the gastric electrical activity is quite stable with slight differences in power ratio and emptying at given recording days. On the contrary, intestinal permeability showed a persistent improvement over the first week of postnatal life.

A few studies have investigated the gastric motility and intestinal permeability in preterm newborns. We studied the gastric electrical activity, gastric emptying and intestinal permeability in a time series in order to account for the effect of the different physiological variables over time. The pattern of slow wave percentage in the normal neonates showed a stable 3 cpm activity over time. During the first month of life the slow wave percentage was usually reported to be about 38% [24] from birth to 4 weeks, whilst according to others the slow wave percentage was about 50% [25]. Our data from premature newborns showed a higher percentage of normal slow wave, probably as a result of our broad interval in the normal EGG frequency ranges.

Intestinal immaturity is limited largely to infants of less than 34 weeks gestation but may extend to older gestational ages. Intestinal immaturity could explain poor gastroduodenal coordination and excessive quiescence in motor activity reported in very immature infants as poor gastric emptying, duodenogastric reflux and gastroduodenal hypomotility [26,27]. Our group of healthy newborns were of about 34 weeks gestation and showed a normal EGG parameters and gastric emptying time, even if subtle differences between the recording days were found. These findings confirm that gastric development is complete in late preterm infants [28-30].

Different sugar-absorption tests for measuring intestinal permeability for sugars have been studied in a variety of gastrointestinal diseases. In vivo mannitol is absorbed via the transcellular pathway and serves as a marker of transcellular uptake [22,23] while lactulose is only slightly absorbed, but exclusively across the intestinal membrane through the intercellular junctions, and serves as a marker for mucosal integrity [31]. In our study L/M ratio was sharply reduced at day 7, then it remained stable. The clinical significance of an increased intestinal permeability is still under investigation. Although alterations in intestinal permeability could cause bacterial translocation and septic complications, no evidence is reported in humans to support this assumption [32,33]. A close relationship between luminal factors and permeability was demonstrated only for IgA, ovoalbumin, and bacterial peptides [34-36]. Overall, the human neonate shows a developmental pattern of sugar intestinal permeability that resembles gut closure observed in other mammals; intestinal permeability decreases faster in breast-fed newborns than in those fed with adapted or hydrolysed formula [37,38]. However, both decreased and increased permeability during the first months of life have been reported [14,39,40]. The reasons for such discrepancies lie in the differences in study design such as gestational age, clinical condition, feeding regiments and postnatal age at the time of the studies. Our data are similar to that of Van Elburg, actually preterm newborns permeability is higher during the first 2 days of life than up to 6 days later, independently of birth weight and gestational age [41]. Our data showed a slight increase in mannitol permeability in day 7 and a dramatic reduction of L/M ratio between day 3 and day 7 related to reduced lactulose permeability. Even if the relationship between feeding and intestinal maturation was not studied in our paper, some authors have demonstrated that the starting of enteral feeding induces an increase in intestinal barrier function [42]. The fact that adult patients fed with total parenteral nutrition showed an impaired intestinal permeability confirms the link between enteral nutrition and permeability [43].

In conclusion, healthy late preterm newborns showed mature EGG and gastric emptying and a rapid improvement in intestinal permeability. The role of enteral nutrients is not merely linked to nourishing the developing intestine of the premature infants but may represent a kick off point. Optimization of nutrition in preterm infants could have major implications for health and outcome.

## Abbreviations

EGG: Cutaneous electrogastrography; GE: Gastric emptying; SAT: Sugar absorption test; DF: Dominant frequency; SW: Slow waves; PR: Power ratio; FFT: Fast Fourier transform; T1/2: Half emptying time.

## Competing interests

The authors declare that they have no competing interests.

## Authors' contributions

GR conceived of the study, carried out the recording of the gastric electrical activity and drafted the manuscript. FI conceived of the study, and carried out the permeability tests and polish the manuscript. RF participated in the design of the study. OM carried out the permeability tests and gastric emptying studies. GS participated in the design of the study. MB performed the statistical analysis. LC participated in the coordination of the study. RF participated in the coordination of the study and polish the manuscript.
